# Draft genome sequences of *Stenotrophomonas maltophilia* strains UPSm1, UPSm3, and UPSm5 and *Stenotrophomonas pavanii* strain UPSm2

**DOI:** 10.1128/mra.00313-25

**Published:** 2025-06-30

**Authors:** Brandi L. Cobe, Egon A. Ozer, Sophia H. Nozick, Alan R. Hauser, Nicholas P. Cianciotto

**Affiliations:** 1 Department of Microbiology-Immunology, Northwestern University Medical School, Chicago, Illinois, USA; 2 Division of Infectious Diseases, Department of Medicine, Northwestern University Medical School, Chicago, Illinois, USA; Wellesley College, Wellesley, Massachusetts, USA

**Keywords:** *Stenotrophomonas*, *Stenotrophomonas maltophilia*, *Stenotrophomonas pavanii*

## Abstract

We report the draft genome sequences of *Stenotrophomonas maltophilia* strains UPSm1, UPSm3, and UPSm5 and *Stenotrophomonas pavanii* strain UPSm2, all of which had been isolated from the respiratory tract of human patients. The genome assemblies contain single contigs that have an average length of 4,864,360 bp.

## ANNOUNCEMENT

*Stenotrophomonas maltophilia* is a multi-drug-resistant pathogen associated with lung infections (1). Within the *S. maltophilia* complex, *Stenotrophomonas pavanii* has rarely been isolated from humans (2). Isolated at the University of Pennsylvania Hospital in Philadelphia ~15 years ago, UPSm1 was obtained from a tracheal aspirate, whereas UPSm2 was isolated from sputum and UPSm3 and UPSm5 from respiratory sinuses (3, 4). The strains had been isolated by plating (without dilution) onto MacConkey agar and incubating at 37°C. They were provided to us without any patient-identifying information and previously provisionally identified by 16S rRNA sequencing, using primers first described by Hauben et al. (4, 5). Since these strains exhibit virulence (3, 4, 6, 7), we determined their genome sequences.

Using the Promega Maxwell 16 system, DNA was extracted after inoculating a colony into Luria-Bertani broth and incubating ~16 h at 37°C with shaking. Sequencing libraries were prepared using the plexWell kit (seqWell) and sequenced on the Illumina MiSeq using a 600-cycle V3 kit (Illumina) to yield paired-end 300 bp reads. The read numbers were 1,860,604 totaling 532,173,911 bp for UPSm1, 1,832,198 totaling 523,499,569 bp for UPSm2, 1,774,132 totaling 502,041,083 bp for UPSm3, and 3,204,674 totaling 917,921,896 bp for UPSm5 after adapter trimming. Reads were quality trimmed, and adapter sequences were removed using fastp (8). Genome sequences were assembled using SPAdes v3.9.1 (9). Residual adapter contamination was identified using National Center for Biotechnology Information (NCBI) FCS-adaptor v0.5.4 (http://github.com/ncbi/fcs) and removed. Assembly contigs shorter than 200 bp or those with an average coverage of <5 reads were removed. Sequences were annotated through the NCBI Prokaryotic Genome Annotation Pipeline version 6.9 (10–12). Software default parameters were used throughout.

The UPSm1 sequence contains 73 contigs having an N_50_ of 249,577 bp. It is 4,911,780 bp with ~73X coverage having a G + C content of 66.24% and was predicted to have 4,469 ORFs. The UPSm2 sequence contains 64 contigs having an N_50_ of 211,717 bp. It is 4,805,651 bp with ~72X coverage having a G + C content of 66.96% and was predicted to have 4,349 ORFs. The UPSm3 sequence contains 40 contigs having an N_50_ of 346,781 bp. It is 4,999,821 bp with ~69X coverage having a G + C content of 66.47% and was predicted to have 4,563 ORFs. The UPSm5 sequence contains 33 contigs having an N_50_ of 452,378 bp. It is 4,740,187 bp with ~127X coverage having a G + C content of 66.08% and was predicted to have 4,318 ORFs. A tetra correlation search, using JSpeciesWS, provided genomes that were most closely related based on their tetra-nucleotide signature correlation index (13). Pairwise average nucleotide identity comparisons (13–15) confirmed that UPSm1, UPSm3, and UPSm5 are *S. maltophilia* and UPSm2 *S. pavanii* (Table 1).

**TABLE 1:** Average nucleotide identity (ANI) values from pairwise comparisons

Pairwise average nucleotide identity compared to strain UPSm1			
Genome	Strain	Accession No.	% ANI
*Stenotrophomonas maltophilia*	EPM1	PRJNA165731	97.64
*Stenotrophomonas maltophilia*	K279a	PRJNA30351	97.32
Pairwise average nucleotide identity compared to strain UPSm2			
Genome	Strain	Accession No.	% ANI
*Stenotrophomonas pavanii*	DSM 25135	PRJNA284376	98.21
*Stenotrophomonas pavanii*	LMG 25348	PRJEB16042	98.14
Pairwise average nucleotide identity compared to strain UPSm3			
Genome	Strain	Accession No.	% ANI
*Stenotrophomonas sp*.	SKA14	PRJNA19369	99.72
*Stenotrophomonas maltophilia*	B5	PRJNA285410	95.65
Pairwise average nucleotide identity compared to strain UPSm5			
Genome	Strain	Accession No.	% ANI
*Stenotrophomonas maltophilia*	LMG 22072	PRJNA299451	97.33
*Stenotrophomonas maltophilia*	OC194	PRJNA295129	92.63

## Data Availability

Projects for UPSm1, UPSm2, UPSm3, and UPSm5 are deposited at DDBJ/ENA/GenBank under accession numbers JBJMAF000000000, JBJMAE000000000, JBJMAD000000000, and JBJMAC000000000, respectively. The versions described here are version 1. Raw reads were submitted under Bio Project accession number PRJNA1180531, with Bio Sample numbers SAMN44521699, SAMN44521700, SAMN44521701, and SAMN44521702. Raw reads were submitted to the NCBI SRA under accession numbers SRS24784622, SRS2478462, SRS24784624, and SRS24784623.
